# Laser Cutting: A Review on the Influence of Assist Gas

**DOI:** 10.3390/ma12010157

**Published:** 2019-01-06

**Authors:** Antonio Riveiro, Félix Quintero, Mohamed Boutinguiza, Jesús del Val, Rafael Comesaña, Fernando Lusquiños, Juan Pou

**Affiliations:** 1 Department of Applied Physics, University of Vigo, EEI, Lagoas-Marcosende 9, 36310 Vigo, Spain; fquintero@uvigo.es (F.Q.); mohamed@uvigo.es (M.B.); jesusdv@uvigo.es (J.d.V.); flusqui@uvigo.es (F.L.); jpou@uvigo.es (J.P.); 2 Department of Materials Engineering, Applied Mechanics and Construction, University of Vigo, EEI, Lagoas-Marcosende, 36310 Vigo, Spain; racomesana@uvigo.es

**Keywords:** laser cutting, assist gas, nozzles, shock waves

## Abstract

Assist gas plays a central role in laser fusion cutting. In this work, the aerodynamic interactions between the assist gas and the workpiece are reviewed. An insight into those phenomena that hinder the cutting quality and performance is provided. These phenomena include shock waves, choking, boundary layer separation, etc. The most relevant and promising attempts to overcome these common problems related to the gas dynamics are surveyed. The review of the current scientific literature has revealed some gaps in the current knowledge of the role of the assist gas dynamics in laser cutting. The assist gas interactions have been investigated only under static conditions; and the dynamic interaction with the molten material on the cutting front has not been addressed. New nozzle designs with improved efficiency of molten material removal are required to improve cut quality; and cutting speed in current industrial laser cutting machines; especially in those assisted by new high-brightness laser sources.

## 1. Introduction

Laser cutting was one of the first industrial applications of lasers, and one of the most widespread in the manufacturing industry since the birth of laser technology. The first reported attempt to use a laser as a cutting tool may be well attributed to P. Houldcroft, who in 1967 used a 300 W CO_2_ laser with oxygen as assist gas to cut 1 mm thick steel sheet [1]. Currently, this kind of processing is routinely applied to cut steels, ranging from 0.5–30 mm in thickness for different purposes, as well as other engineering materials. The high material versatility, edge quality, easy automation and operation, accuracy and production flexibility in combination with a high material utilization and virtually no tool wear, are just some of the advantages over other conventional and non-conventional cutting methods. Laser cutting machines are mainly used to shape, cut, bore, drill, or tap metal components. Many different industries currently apply laser cutting, including the automobile, aerospace, medical devices, renewable energy, semiconductors, and consumer electronics industries.

Consuming approximately 25% of the total energy in Europe [2], the manufacturing industry is responsible for a substantial part of the total environmental impact. Furthermore, this impact is expected to increase, taking into consideration the current trend towards more energy intensive processes [3]. One would think that laser cutting had reached its maximum efficiency; however, the process has not been pushed to its limits, and it still has the potential to be tuned to a greater extent than at present.

Steen pointed out six different mechanisms for laser assisted cutting of materials, namely, vaporization cutting, melting and blowing, burning and blowing, thermal stress cutting, scribing and cold cutting [4]. Laser cutting techniques based on melting, burning, and blowing are mostly used in industry [5]. These are suitable to process materials such as metals, thermoplastics and some glasses and ceramics. They involve the localized fusion of the workpiece with a focused laser beam, and the utilization of an inert or reactive assist gas to sweep the molten material. If the gas jet is chemically inert in contact with the molten material, the process is called laser fusion cutting. When a reactive gas is used to assist the process, it is designated as laser reactive fusion cutting. Both processes are used for sheet metal cutting, which is the largest industrial application of laser cutting machines. Despite the simplicity of the process, multiple parameters affect the result in terms of productivity or quality. Table 1 summarizes the main processing parameters in laser fusion cutting, and laser reactive fusion cutting of any material. They are related to the laser beam, to the beam guidance, assist gas properties, and transport properties. For a given material, some of them contribute to the melting (those related to the laser beam, beam guidance, transport properties, and even some related to the assist gas if this can exothermically react with the material), while others contribute to the removal of this molten material (those related with the assist gas). Current research mainly focuses on real-time process monitoring and control [6], and on increasing the laser source efficiency [7] as a method to enhance the melting of the material. In this sense, high power fiber and disk lasers have been introduced during last decade as an alternative to the traditional CO_2_ laser sources. Disk and fiber lasers have a higher wall-plug efficiency, and better beam absorption behavior in metal sheets. The better beam absorption allows one to achieve very high processing rates in the cutting of thin metal sheets; however, at higher thicknesses (typically over ~4 mm), the surface quality is much worse compared to the CO_2_ technology [8]. Therefore, secondary operations, such as deburring, can be required in these cases. This indicates that the success of the process does not only depend on melting as much material as possible, because this molten material must also be extracted at the same rate. Both processes, melting and blowing, must be balanced; the preponderance of one of them will make the process nonviable or significantly reduce the quality of the resulting part. Therefore, those parameters related to the assist gas must also be optimized to enhance the overall efficiency of the whole process.

Due to the importance of the assist gas, many works dealing with its different roles in laser cutting can be found in the literature. Some of them study the aerodynamics of the assist gas jets, others focus on the interaction of the jets with workpiece, others on the influence of the nature and purity of the gas, etc. In this paper, we have reviewed the most relevant works, and most recent findings on the fluid mechanic mechanisms involved in laser fusion cutting. Works on the role of the assist gas in laser cutting can be categorized as follows:

- Study of the gas jets emerging from converging nozzles.
- Study of the interactions of the gas jet emerging from converging nozzles with the surface of the workpiece.
- Study of the interactions of the gas jet with the cut kerf.
- Study of the interactions of the gas jet with the molten material into the cut kerf.
- Study of different nozzles or solutions to solve problems found in the interactions of the gas jet during laser cutting.

These topics will be addressed in the following sections.

## 2. Removal Mechanisms of Molten Material

Olsen determined the maximum cutting rate for different thicknesses of a given workpiece as a function of the laser power and the assist gas pressure [10]. He used a theoretical model specifically developed for laser cutting simulation [11]. The main finding of this work is that the limiting factor affecting maximum cutting rate is the force exerted by the assist gas inside the cutting kerf instead of the laser power. This highlights the relevance of the gas in the process. Furthermore, these limitations are especially becoming important when thick sections are processed [12]. Vicanek and Simon developed a model which demonstrates that these forces determine the melt thickness on the cutting front, and the cutting speed [13]. If these forces are weak, accumulation of melt on the cutting front is observed. Moreover, a worsening on the cut quality occurs when an excessive amount of molten material is accumulated into the kerf [14].

**Figure 1:**
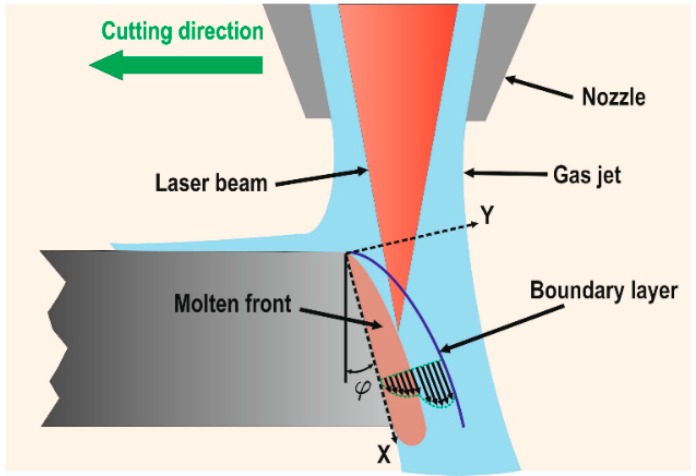
Scheme of the forces acting on the molten material during laser fusion cutting.

When the assist gas is flowing into the kerf, a boundary layer is developed (see Figure 1). The momentum transfer from the assist gas to the molten material is carried out through this boundary layer. In order to maximize the transference of momentum to the molten material, the boundary layer must be kept into the laminar regime. This requirement is fulfilled for Reynolds numbers (Re) of the flow less than a critical value (Re_g_ < Re_g,crit_ = 3.2 × 10^5^). When Re_g_ > Re_g,crit_ the flow turns from laminar to turbulent. This point is called separation point or boundary layer separation (BLS).

Vicanek and Simon theoretically investigated the forces exerted by the assist gas on the molten layer and developed a dynamical model of the melt ejection in laser cutting [13]. These forces were identified as the pressure gradient along the cutting front and the shear stresses generated by a viscous friction of the assist gas on the molten material (see Figure 1). They also provided an estimation for the value of these forces. For cutting thin sheets, a laminar gas flow inside the kerf is supposed because the short length of the kerf prevents the mixing of the jet with the surrounding atmosphere. The nature of the boundary layer was considered laminar because the Reynolds number is three orders of magnitude below the critical value. The gas flow into the kerf is split in two regions, an inviscid region and a boundary layer between the gas and the molten material. Under these main assumptions, scaling expressions for the gradient pressure and the shear stress were derived using conformal mapping techniques, and boundary layer theory,

(1) ∂p∂x=−(p0d)a(xd,φ);p0=12ρgUg2

(2) τ=τ0b(xd,φ);τ0=(μgρgUg3d−1)12

being *a* and *b* non-dimensional functions, *x* the distance along the cutting front, *d* the nozzle diameter, φ the inclination of the cutting front, ρ_g_ and *U*_g_ the density and velocity of the gas, and µ_g_ the gas viscosity. From these equations, it is deduced that both forces are of the same order of magnitude, and both increased with the gas velocity. Other authors also pointed out that these forces depend on the gas velocity and density [15].

Analytical expressions to calculate these forces were proposed by other authors using the common drag equation, but using different friction coefficients. The shear stress can be estimated as [16],

(3) $$\tau =\frac{C_{f}}{2}\rho_{g}U_{g}^{2}$$

being *C*_f_ the local skin friction coefficient at the gas-liquid interface (Cf=0.0576(Reg)−1/5, and Re_g_ the Reynolds number of gas flowing over the molten metal). The shear stress acting on the molten metal due to the gas jet can be assumed as [17],

(4) $$\tau =\frac{f}{4}\frac{\rho_{g}U_{g}^{2}}{2}$$

being *f* the friction coefficient for a turbulent boundary layer in a smooth pipe:

(5) 1f~2log(Regf2.5)

The difference between Equations (3) and (4) arises from the estimation of the friction coefficient. 

Kaplan estimated the force due to pressure gradient and shear stress using equations to calculate the force due to a static and dynamic pressure [18]. They are split into a normal (*F*_0_ and *F*_n_ due to the static and dynamic pressure respectively), and tangential force (*F*_t_ due to shear stress) to the cut kerf:

(6) $$\begin{array}{c} F_{0}=dw\frac{\pi }{2}p_{g}\\ F_{n}=dw\frac{\pi }{2}\rho_{g}U_{g}^{2}\tan\varphi \\ F_{t}=\sqrt{d}w\frac{\pi }{2}\sqrt{\rho_{g}\mu_{g}}2U_{g}^{3/2} \end{array}$$

where *w* is the kerf width, and *p*_g_ is the reduced gas pressure at the exit (due to the sideward expansion of the gas after exiting the nozzle).

The density and viscosity of the assist gas used in the process also plays a role on the removal of molten material. As deduced from Equations (3)–(5), the removal action of the gas is larger for high Reynolds number of the gas flowing over the melt. This a dimensional number can be written as:

(7) Reg=ρgUgwμg

being ρ_g_ and µ_g_ the density and viscosity of the assist gas, *U*_g_ the velocity of the gas jet and *w* the width of the cut kerf (commonly, ranging from *w* = 0.1 up to 0.5 mm). It is worth noting that assist gases with a larger density (e.g., argon) are more efficient than others (e.g., helium) because the Reynolds number is promoted [19].

From the precedent analysis on the efforts to calculate the forces involved in the removal of molten material by the assist gas, several conclusions can be drawn. First, all of the works agree that the removal of molten material is promoted by increasing the velocity of the gas jet; however, none of them take into account the compressibility of the assist gas. Furthermore, it is assumed that the assist gas flow is laminar, an oversimplification taking into account the large velocities involved, and the interaction of the gas with the workpiece. However, these models do not entirely capture the physics of the process, and strong deviations of predictions can occur. Effects related to these topics will be addressed in the following sections.

## 3. Aerodynamic Interactions during Laser Cutting

**Figure 2:**
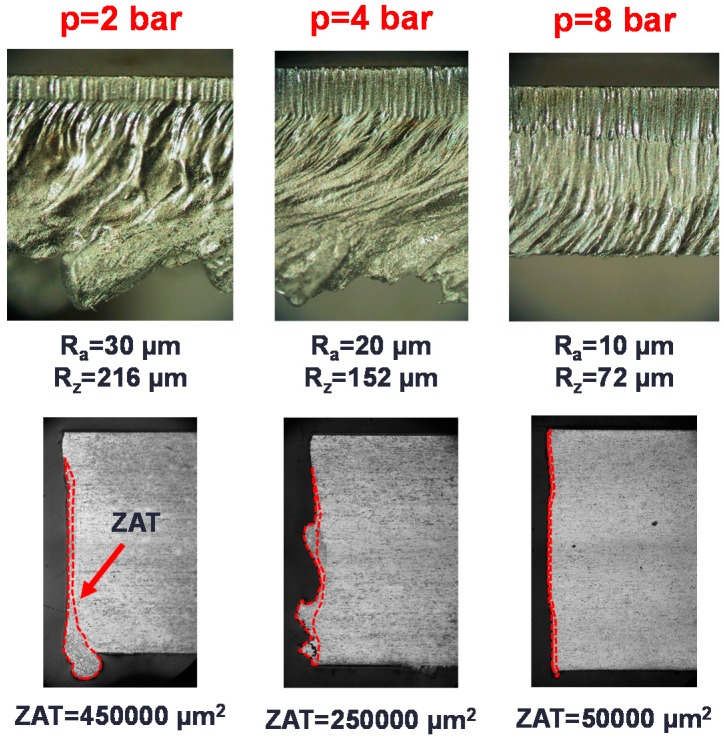
Cutting edge and cross section of aluminum-cooper alloy samples (2024-T3) processed by means of a CO_2_ laser using a conventional cutting head working with three different supplying pressures (Processing parameters: Laser power *P* = 2500 W, cutting speed *v*_c_ = 4000 mm/min, focal length *f* = 127 mm, stand-off *Z* = 1.5 mm, conical nozzle, nozzle diameter *d* = 2 mm, assist gas: argon).

As deduced from Equations (1)–(6), the removal forces performed on the molten material are promoted with the velocity of the assist gas. It is customary to increase the supply pressure to promote this effect, however, this can lead to unsatisfying quality results as seen in Figure 2. As observed, a threshold gas pressure is required to avoid the formation of dross, however, some resolidified material and striations along the cutting edge cannot be avoided. A further increment of the gas pressure does not significantly improve this edge quality. For some engineering applications, this quality level is acceptable, but some challenging applications (e.g., aerospace applications) may require higher cut quality levels. The uncompleted removal of molten material and the reduced cut quality can be explained due to the compressibility of the assist gas as explained in this section.

### 3.1. Free Jets from Converging Nozzles

**Figure 3:**
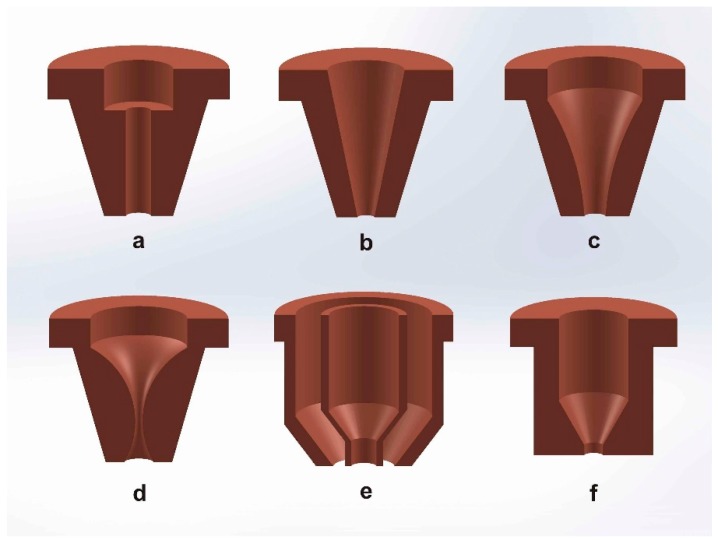
Nozzle geometries commonly used for laser fusion cutting: (**a**) parallel, (**b**) conical, (**c**) converging, (**d**) converging-diverging, (**e**) annular, and (**f**) flat tipped. Reprinted with permission from [22]; Copyright 1986 SPIE.

The most relevant parameters during CO_2_ laser cutting, which affect the quality of the cut, are laser power, feed rate, metal thickness, nozzle design, and the gas used in the jet [20]. Nozzle design was identified as a critical parameter affecting the cutting performance during laser fusion cutting, because it directly affects the gas flow characteristics [21]. Fieret et al. performed the first serious attempt to ascertain the role of the assist gas in laser cutting [22]. As they noticed, common gas nozzles used in laser cutting have a convergent internal geometry (see Figure 3a–c,e,f ) to supply the gas coaxially regarding the laser beam; indeed, conical nozzles are the largest class in the industry. The gas jet emerging from these designs (except for the nozzle d in Figure 3) is subsonic or transonic. Different nozzle designs (see Figure 3) give different static pressures, assist gas velocities, and consequently different cut quality levels.

Although the behavior of the free jet is different during the processing, the study of the free jet emerging from a nozzle will approximately indicate its subsequent performance during laser cutting. Using a pressure transducer, the effective pressure along the assist gas jet ejected by a conical nozzle was measured for different supplying pressures [22]. A strong variation of the effective pressure was observed as a function of the supplying pressure, especially for pressures higher than 4 bar. Man et al. suggested that this non-uniformity of the effective pressure along the jet would result in poor and inconsistent cut quality, a low cutting speed and high wastage of cutting gas [23].

As known from the gas dynamics theory, when a gas passes through a converging nozzle the gas is expanded, i.e., the velocity of the gas increases and the pressure decreases. Converging nozzles can only expand the gas up to sonic regimes. When this occurs, the nozzle is said to be “choked” and the Mach number at the exit of the nozzle is *M* = 1. The ratio supplying/ambient pressure (*p*_e_/*p*_a_) (also called underexpansion ratio) is a key parameter defining the evolution of the jet after exiting the nozzle [24]. The jet is referred to as being underexpanded if *p*_e_/*p*_a_ > 1, overexpanded if *p*_e_/*p*_a_ < 1, and pressure matched if *p*_e_/*p*_a_ = 1 (being *p*_e_ the pressure just at the exit of the nozzle). As a rule, jets used in laser cutting are underexpanded, i.e., their static pressure is higher than ambient, because the supplying pressure is commonly greater than 2 times the ambient pressure. Common pressures used in laser cutting range from *p* = 2–3 up to *p* = 20 bar, except during the processing of mild steel with oxygen where lower pressures are used to minimize side burning, especially in cutting thick sections.

**Figure 4:**
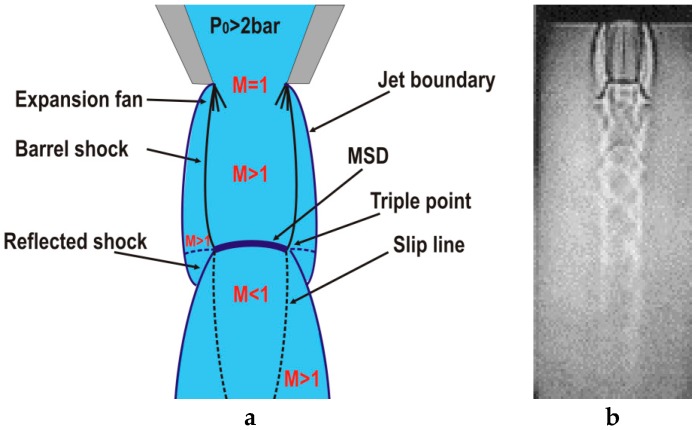
(**a**) Scheme and (**b**) shadowgraph image of the free jet emerging from a conical nozzle commonly used in laser fusion cutting for an operating parameter range *p*_e_/*p*_a_ > 1.89. Adapted with permission from [23]; Copyright 1998 Elsevier.

Underexpanded jets display a complicated axisymmetric structure (see Figure 4). The jet boundaries oscillate because the gas is periodically overexpanded, and then converge in an attempt to match the ambient pressure. This matching takes place by means of shock wave phenomena. Assist gas leaving the nozzle tries to expand because emerges at a pressure lower than ambient. The expansion waves from the nozzle reflect from the constant pressure streamline (jet boundary) as compression waves, subsequently coalescing to form the barrel shock. Depending on the flow conditions, the barrel shock may reflect regularly at the centerline (moderated underexpanded jets), or it may terminate in a triple point (highly underexpanded jets). In laser cutting, highly underexpanded jets are normally used and a strong normal shock wave called Mach Shock Disk (MSD) is formed, as depicted in Figure 4. Behind the Mach disk, a region of subsonic flow (*M* < 1) bounded above by a slipstream (slip line) emanating from the triple point, appears. Leidinger et al. used a finite volume code to simulate the free stream that emerged from an underexpanded conic-cylindrical nozzle [25]. They determined that after crossing the MSD, the velocity and energy of the gas is reduced, and the stagnation pressure on the workpiece decreases (the reduction in pressure on the workpiece was first observed by Fieret et al. [22]). These effects are negative because the removal efficiency of the assist gas is closely related to the velocity of the jet (as given by Equations (1)–(6)).

The Mach disk location (*x*_M_), can be determined by the following Equation [26],

(8) $$\frac{x_{M}}{d}=0.67\sqrt{\frac{p_{0}}{p_{b}}}$$

being *d* the nozzle diameter, *p*_0_ the stagnation supplying pressure and *p*_b_ the background pressure (i.e., the atmospheric pressure, *p*_b_ = *p*_a_, for common working conditions where vacuum a is not applied). Note that this equation is independent of the nature of the assist gas (i.e., independent of heat capacity ratio γ).

**Figure 5:**
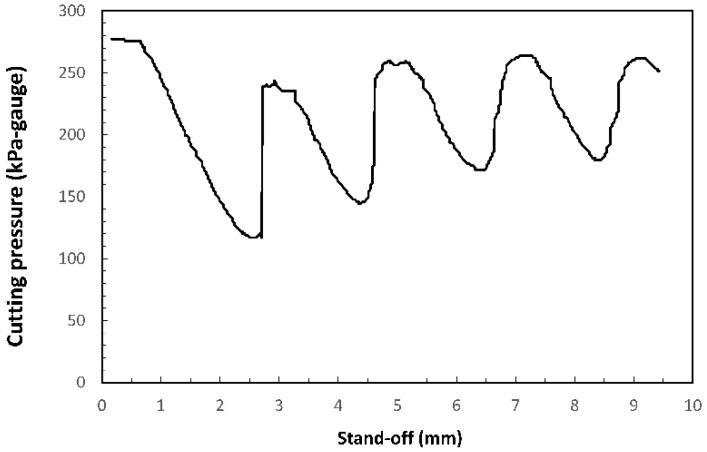
Variation of the pressure along the jet (air, conical nozzle, nozzle diameter *d* = 1.5 mm, gauge pressure *p* = 280 kPa) when the workpiece is moved away with regard to the laser beam. Reprinted with permission from [27]; Copyright 1984 ICALEO.

In summary, large variations of the properties (pressure, velocity, density, etc.) are found in the free jet as a consequence of these shock phenomena. In order to quantify these variations, Ward, using a pressure transducer, measured the pressure variation on the workpiece along the centerline of the jet (see Figure 5), and also radially as a function of the supplying pressure and the stand-off [27]. The aim was to determine the effective pressure exerted on the workpiece surface. He showed that some combinations of assist pressure and stand-offs maximize the pressure of the assist gas on the workpiece, but others minimize this parameter. The formation of the first Mach Shock Disk (MSD) was estimated to occur at a distance from the nozzle around 2 mm (decrease in cutting pressure observed at around 2 mm in Figure 5). Man et al. obtained similar results theoretically [23]. They numerically showed an increment of the centerline pressure after crossing normal shocks (MSD) formed into the jet. In turn, thrust followed an opposite behavior. At first, it increased, but after crossing the MSD, it decreased. Centerline momentum was seen to reach a maximum for distances around x/d≈0.46 from the exit of the nozzle (being *x* the distance from the nozzle tip, and *d* the exit diameter of the nozzle). The minimum momentum decreases when the supplying pressure is increased, indicating a low tolerance of cut quality to variations in the stand-off distance.

Man et al. also computed the centerline pressure, thrust, and shape of jets from subsonic and transonic nozzles [23]. A non-uniformity of the axial thrust due to the shock wave pattern in the flow caused strong fluctuations in the gas momentum inside the kerf, leading to a poor ejection of molten material. The tendency to dross formation is higher, and the tolerance of the stand-off is too low. Thus, poor cutting quality and low cutting speed are resulted. Furthermore, they computed and observed the cross-sectional area of the MSD and an increase with the pressure was obtained. Therefore, the increment of the shock strength combined with the reduction in the gas flow entering into the kerf (as showed in Section 3.2) also produced a reduction of the cut quality.

Chen et al. calculated, by means of computational fluid dynamic (CFD) analyses, the variation of the pressure distribution of an underexpanded jet emerging from a conical nozzle [28]. The results were compared with experimental results obtained previously in other works and showed good agreement. Non-uniformity of properties in the jet is greater when supplying pressure is increased. This conclusion was confirmed by means of CFD simulations [29]. The static pressure profile along the axis of a free jet was computed for different supplying pressures. Results indicate that the MSD induces a low-pressure region for assisting pressures higher than 4.7 bar (in the case of nitrogen as assist gas); however, properties for low underexpanded jets (*p* < 4.7 bar) were seen to vary smoothly along the jet.

**Figure 6:**
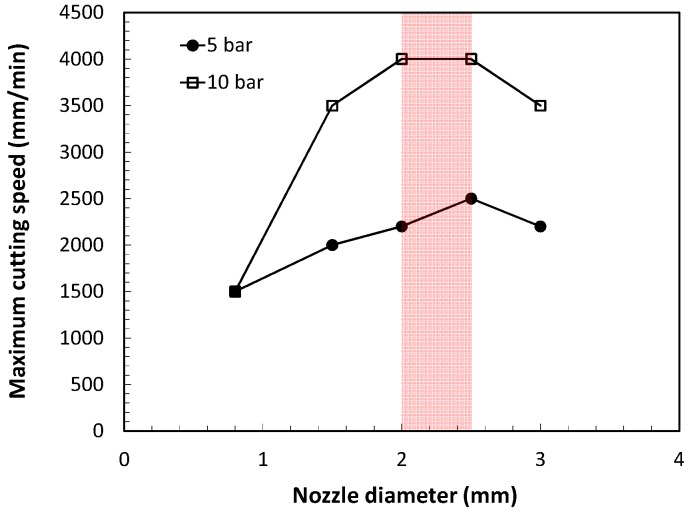
Dependence of the maximum cutting speed on the nozzle exit diameter for two supplying pressures (*p* = 5 and 10 bar) during laser cutting of aluminum-cooper alloys 3 mm in thickness (Processing parameters: Laser power *P* = 2500, stand-off *Z* = 1.5 mm, focal length *f* = 127 mm, conical nozzle, assist gas: argon). Adapted with permission from [30]; Copyright 2010 Elsevier.

The exit nozzle diameter was also found to be a key parameter. Kamalu and Steen pointed out that there is an optimum nozzle diameter to achieve maximum cutting speeds [21]. Utilization of small diameters leads to the location of maximum thrust at shorter *x/d* distances from the nozzle exit. Utilization of larger diameters increases this distance but also the gas consumption. Riveiro et al. measured the maximum cutting speed as a function of the nozzle diameter during laser cutting of aluminum-copper alloys (see Figure 6) [30]. Nozzle diameters in a range from *d* = 2.0 to 2.5 mm are observed to maximize cutting speed.

### 3.2. Gas Jet Impingement. Choking 

In laser cutting, previously discussed phenomena are aggravated in the jet due to the presence of an obstacle, in this case, the workpiece. A decrease in the cutting speed, rather than an increase was observed when the supplying pressure was raised up to high values [21]. Adams proposed that this phenomenon was related to the cooling action of the assist gas on the molten metal [31], but Kamalu and Steen demonstrated that the cooling effect in laser cutting due to the assist gas was negligible [21]. Vicanek and Simon also corroborated that result [13]. They calculated the total cooling rate of molten material at 2300 K, in a cut kerf of 50 µm in width, for a 3 mm thick workpiece, and using a gas jet which emerges at 100 m/s from the nozzle. They obtained 2.5 W of cooling rate, which is negligible as compared to the incident laser power of typically several kW.

**Figure 7:**
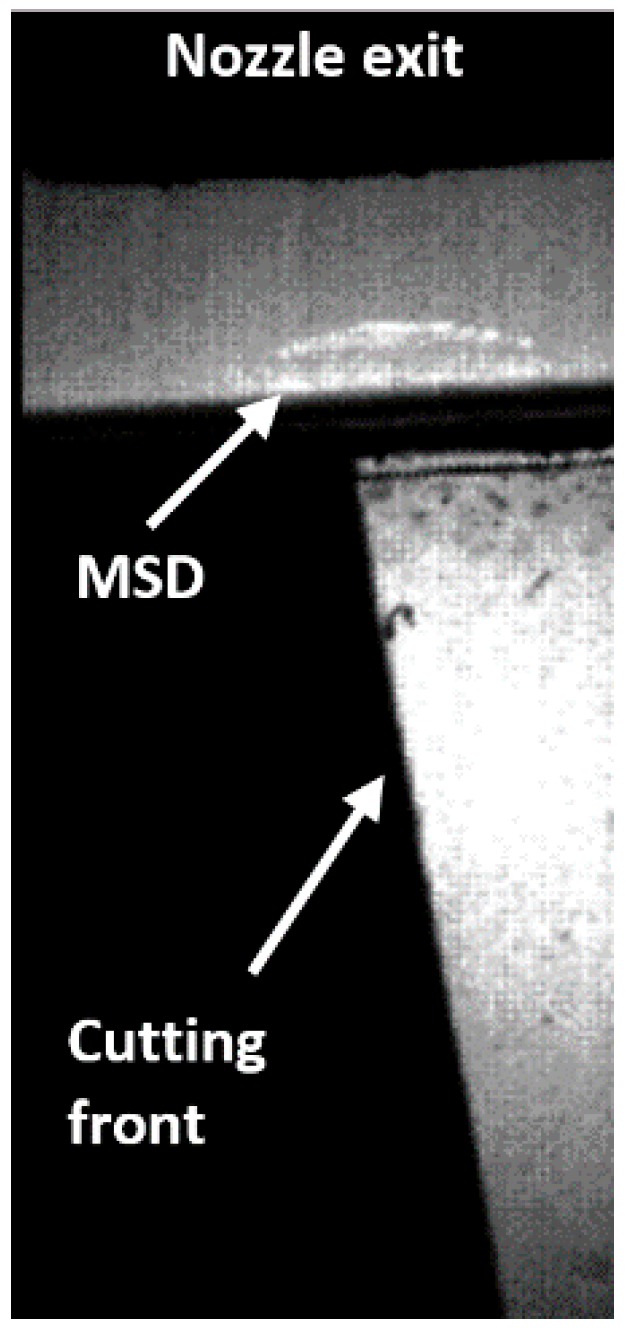
Shadowgraph imaging of the Mach Shock Disk (MSD) formed at the entrance of a simulated cut kerf (Lateral view of the cut slot. Supplying pressure *p* = 8 bar, stand-off *Z* = 1 mm, nozzle diameter *d* = 2 mm).

The presence of shock waves in the flow, and in particular the existence of a density gradient field (associated to the formation of a normal shock wave, MSD) just at the entrance of the kerf (see Figure 7) were the causes of this decrease in cutting speed when cutting at high assist gas pressures [22].

According to La Rocca [32], during the laser cutting process the larger cross-sectional area of the jet, as compared to the kerf dimensions, leads to a high area blockage of approximately 90%. As a consequence, the gas jet suffers a strong choking in the inlet kerf. This blockage is extremely sensitive to an increment in the pressure. In order to avoid choking, he proposed the utilization of nozzles with exit diameters similar to that of the kerf. Furthermore, he proposed the utilization of non-coaxial nozzles with the impacting point of the jet just before the laser beam in order to also reduce the choking [32].

Fieret and Ward noticed using pressure measurements and flow visualization that choking combined with the normal impingement of an underexpanded jet onto the workpiece, reinforces the strength of the normal shock wave (MSD) formed just upstream of the entrance of the kerf, even at underexpansion rates lower than 3–4 [33]. More technical details about this kind of flow can be found in comprehensive studies of the characteristics of impinging jets [34,35]. Lim et al. calculated the stagnation pressure on the workpiece [36]. After crossing the MSD, the Mach number of the flow and the gas pressure decrease rapidly.

**Figure 8:**
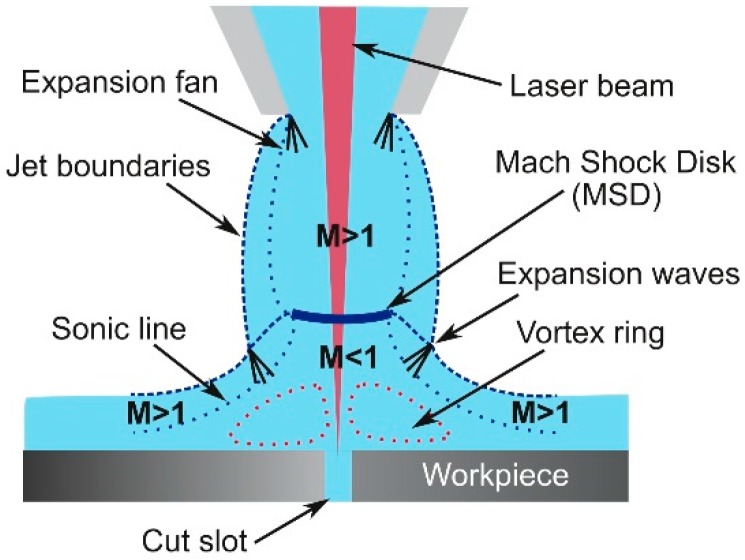
Gas flow structure after exhausted by a conical nozzle just at the entry of the cut kerf.

Fieret et al. sketched the main characteristics of a highly underexpanded jet impinging onto a surface [22]. They observed that prior to entering the kerf, the slipstream and the radial diverging flow in the jet generate a stable vortex ring just inside the inlet kerf as depicted in Figure 8. Carling and Hunt deduced a similar pattern measuring pressures after the MSD and using an indirect method [35]. This consisted in the impingement of a jet for a short time on a layer of a highly viscous mixture of water-pump grease and lamp-black applied to the surface of a flat plate. It was observed that the usual central stagnation point is transformed into a ring surrounding the separated region. Kovalev et al. studied the assist gas flow pattern into the kerf solving the Navier-Stokes equations, using a finite-difference scheme, for a computational domain resembling the cut kerf [37]. Results were compared with the visualization of a thin film of liquid into a simulated cut kerf. They observed the presence of these vortexes during the assist gas performance. In addition, these authors noted that vortexes could trap ambient air into the assist gas. Ivarson et al. [38,39], after experimental and theoretical investigations during laser cutting of mild steel with oxygen, pointed out that this will lead to a decrease of the purity of the assist gas, and ultimately to the degradation in the cut quality during laser cutting of steel using oxygen. O’Neill and Steen proposed to use a peripheral oxygen jet to protect the main gas jet and avoid the contamination of the oxygen jet used in laser cutting of steels [40]. Marginal improvements were achieved during the processing of 3–10 mm steel plates; however, some improvements were observed for thick plates (16–20 mm). Probably, impurities are a consequence of the vortex formation in this case, due to the low influence of compressibility effects (as the supplying pressures ranged from 1.5 to 1.7 bar).

Grigoryantis noticed that the formation of this vortex also increases the residence time of the assist gas in the interaction region, mixing it with vaporized material from the kerf [41]. This fact increases the tendency to plasma formation. The combination of precedent effects can substantially modify the melt removal rate.

The gas decelerates across the MSD to subsonic values and the pressure rises above the ambient pressure [28] (see Figure 8). On the other hand, this shock wave increases the cross section of the jet, and the problem of choking is aggravated. Several parameters have a strong influence on the formation of the MSD and the increment of choking, namely, stand-off distance, kerf width, exit diameter, and supplying pressure.

Riveiro et al. noted that the stand-off distance is a relevant parameter affecting the final cut quality of a laser processed workpiece [30]. They observed the influence of this parameter on cutting speed and quality during CO_2_ laser cutting of an aluminum-copper alloy, in particular on the amount of dross and roughness of the cut walls. Caristan recommends keeping this parameter at 90% of the nozzle diameter [42]. Stand-off distance is monitored and controlled during industrial practice by using distance sensors, such as capacitive sensors (used with conducting workpieces), or “tactile” (contact) height sensors (for insulating workpieces). Leidinger and Schuöcker found the extreme dependence of cut quality with the stand-off distance for highly underexpanded jets by means of numerical simulations and Schlieren photographs [43]. They also computed the pressure distribution of underexpanded jets emerging from converging nozzles, finding a maximum for a stand-off distance of 1.5 mm, i.e., when the workpiece is just before the position of the first Mach shock disk. When the stand-off distance is close to the first MSD, the maximum pressure on the workpiece is reduced. Visualization of the flow by means of Shadowgraph technique performed by Man et al. gave an insight into the influence of the stand-off distance on the MSD formation [44]. When the stand-off distance is zero, the MSD is not produced because the jet is expanded only into the kerf. This situation is not realistic because the tip of the nozzle would be damaged during the process. If the stand-off distance increases, the jet is radially expanded, the centerline pressure of the jet decreases and the choking increases. Kerf width also affects the flow pattern before entering into the kerf. When kerf width is reduced (typically ranging from 100–500 µm), most of the gas jet impinges on the workpiece and the MSD is stronger. Fieret and Ward proposed the following empirical relation to find the optimum stand-off distance as a function of the nozzle diameter and assist pressure [33]:

(9) $$L=d\left(0.5+0.89\sqrt{p-90}\right)$$

where *d* is the exit diameter of the nozzle, *p* the supplying gauge pressure measured in kPa, and *L* the distance from the nozzle to the workpiece).

### 3.3. Gas Flow Pattern into the Kerf

Shear stress and gradient pressure exerted by the assist gas on the molten material are strongly dependent on the flow pattern into the kerf. Zefferer et al. (1991) performed one of the first works dealing with the problem of the assist gas flow into the kerf [45]. They visualized the gas flow into a simulated transparent cutting kerf by means of the Schlieren technique. Chen et al., using CFD simulations and experimental results, obtained the flow pattern just after exiting the nozzle and into the kerf [26,46]. They pointed out that the total gas pressure just before the inlet kerf determines the mass flow into the kerf. They also noticed that shear force exhibits the same pattern. This result highlights the intimate linking between flow structure, mass flow, cutting quality, and efficiency. The increment of the mass flow along the cutting front also increases the melt removal rate. They pointed out that under some conditions, namely high assist gas pressure and certain intervals of stand-off distances, oblique shock waves emerging from the nozzle can directly interact with the MSD. Total pressure just after the MSD abruptly decreases as well as the mass flow through the kerf. Shear forces and pressure gradient were observed to experience certain fluctuations under these conditions. They disrupt the molten material removal and the quality is negatively affected. Experimental cutting results confirmed the influence of this shock structure on the cut quality. On the other hand, they pointed out that more favorable operating conditions exist for larger stand-off distances than those usually used in the practice (commonly *Z* = 0.5–1.5 mm).

**Figure 9:**
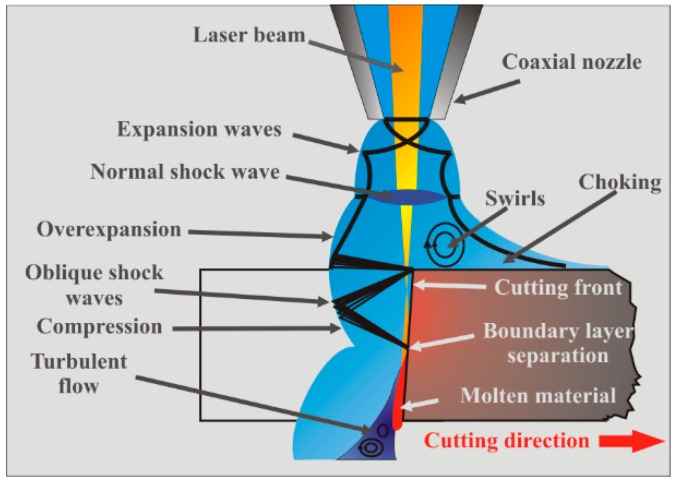
Aerodynamic interactions during laser cutting using a conical nozzle showing the boundary layer separation. Adapted with permission from [30]; Copyright 2010 Elsevier.

**Figure 10:**
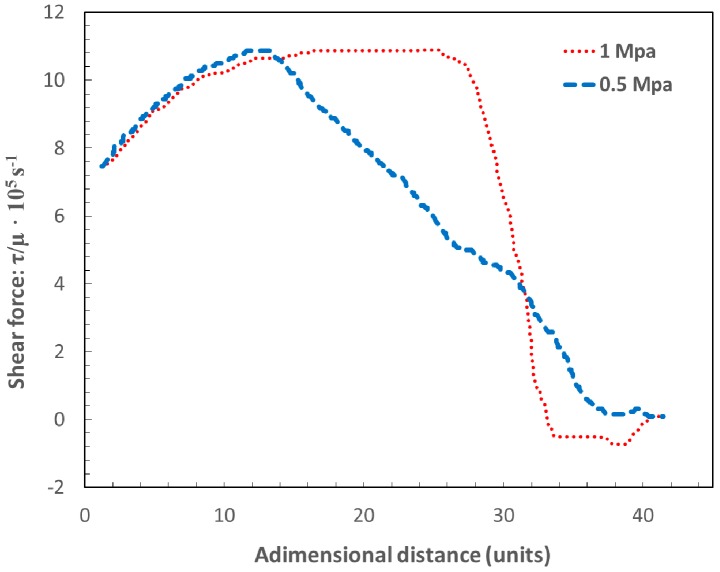
Shear force distribution acting along the cutting front (when using a conical nozzle). A clear drop is noted after the boundary layer separation (estimated shear force in arbitrary units acting on the centerline of the cutting front for *p* = 1 MPa and *p* = 0.5 MPa). Adapted with permission from [49]; Copyright 2001 ICPE.

Makashev et al. [47] noticed that after the normal shock, the flow regimen turns to subsonic (*M* < 1). However, in general, most parts of the flow into the kerf are in the supersonic regimen. When the gas jet enters into the kerf, it is axially and radially expanded in order to match the ambient pressure. The gas jet accelerates, but it meets an oblique shock wave. The reflection of expansion waves at the jet boundaries produce compression waves. Their coalescence lead to the formation of the oblique shock wave shown in Figure 9. Zefferer et al. characterized the principal pattern of the cutting gas flow and its functional dependences from nozzle design and nozzle adjustment, cutting gas pressure and cutting kerf geometry using the Schlieren method [45]. He observed the formation of these compression waves. The impact of this oblique shock wave onto the cutting front imposes a negative gradient pressure on the boundary layer formed by the assist gas and the molten material. Green, in a review of the interactions between shock waves and turbulent boundary layers [48], pointed out that if the strength of this reversed gradient is high enough, it can produce the separation of the boundary layer and its transformation into a turbulent flow. Simulations performed by Leidinger et al. confirmed the boundary layer separation [25]. Horisawa (2001) performed CFD simulations of the assist gas into the kerf and corroborated the results with the flow visualization using the Schlieren method [49]. He noticed an abrupt reduction on the shear force acting on the cutting front after the boundary layer separation, affecting the melt removal rate, and the cutting performance (see Figure 10). The increment of the supplying pressure was seen not having an appreciable effect on the maximum shear force prior to the boundary layer separation. On the other hand, Lim et al. calculated the pressure and shear force distribution on the cutting front [36]. They observed that pressure and shear forces are on the same order of magnitude for low assist gas pressures (as also pointed out by Vicanek and Simon [13]). For higher supplying pressures, the removal of molten material was seen to be primarily due to pressure, while the shear forces are not substantially modified.

**Figure 11:**
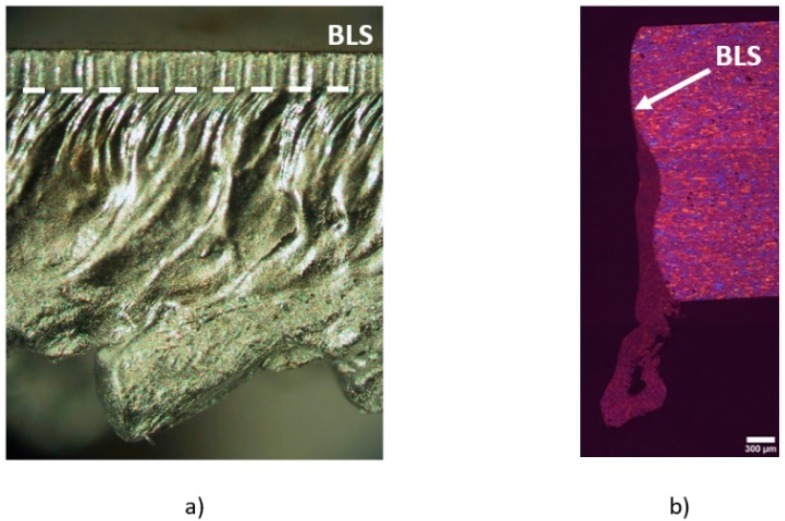
Two regions in the (**a**) cutting edge and in (**b**) the cross section are observed after the boundary layer separation (BLS) during laser cutting of an aluminum-copper alloy, and using a conical nozzle. Reprinted with permission from [30]; Copyright 2010 Elsevier.

Quintero et al. [50] noticed that two zones can be distinguished in the final cutting edge after the boundary layer separation (see Figure 11a): an upper part with low roughness, and a rougher lower part with the presence of a large amount of resolidified material and dross. Detachment of the boundary layer affects both the finishing and the extension of the heat affected zone (HAZ) in the cutting edge (see Figure 11b). HAZ is increased due to the additional release of heat from the solidification of the unremoved molten material after the boundary layer separation.

Several investigations were conducted in order to determine the influence of operating parameters on the boundary layer separation, namely, assist gas pressure, nozzle diameter, angle between laser beam-nozzle, kerf width or sheet thickness. Horisawa et al. showed that the boundary layer separation becomes exaggerated with the increase in assist gas pressure [51]. Man et al. observed that the gas flow into the kerf is better when the assist gas pressure is decreased from 7 to 4 bar [44]. This is because more mass flow penetrates into the kerf, and the gas flows along the kerf without significant divergence.

Horisawa et al. [52] performed the flow visualization by a Schlieren method on a simulated kerf. Total pressure distribution measurement and a CFD (Computational Fluid Dynamics) analysis were conducted. It was observed that positions of flow separations move downward as the pressure increases. The same result was found by Zefferer et al. with the help of the Schlieren method to investigate these gas-dynamical phenomena [45]. Then, a minimum supplying pressure must be applied to locate the boundary layer separation in the exit of the cutting front. Probably, this pressure will be similar to that threshold pressure required to avoid the formation of clinging dross in the cutting edge (as said in Section 3), but no experimental work is found in the literature to support this assertion. The increment of the sheet thickness increases this minimum pressure [45]. The disadvantage of this approach to obtain clean cutting edges is the increment in the gas consumption. Observing the pressure distribution inside the kerf, Horisawa et al. noticed the presence of a peak value before the boundary layer separation [51]. This peak in pressure is slightly increased when the supplying pressure is also increased. The peak is also very sensitive to small stand-off distances or large kerf widths. Larger stand-offs reduce the peak pressure and very low influence is observed when the assist pressure is increased, especially for small kerf widths.

Most of the previously reviewed works, based on flow visualizations or CFD simulations, have not taken into account the influence of the angle of the cutting front on the shock wave structure into the kerf. Kaplan measured the inclination of the cutting front with regard to the laser beam [9]. A value around 1°–15° was obtained. The approach followed by Mai and Lin is the only work giving an insight into the influence of the inclination of the cutting front [29]. It is found that shock waves reflect on the cutting front, giving an asymmetric pressure distribution. When the angle formed by the assist gas jet with the cutting front is small, the maximum pressure on the cut front moves upwards, and the gradient pressure is steep and decreases abruptly along it. Therefore, the maximum rate of molten material removal will be located close to the kerf inlet and will be abruptly decreased along the cutting front.

Other parameters affecting the position of the boundary layer separation are the sheet thickness and the cutting kerf geometry. Boundary layer separation is exaggerated when the kerf width is reduced and supplying pressure is very high. Horisawa showed that the position of this separation tends to be deeper when the kerf width is enlarged as the gas flow is less disturbed [49]. On the other hand, shorter stand-off distances and large kerf widths produce more favorable assist gas flow into the cutting kerf [51]. When the stand-off distance is zero, the gas jet is expanded only into the kerf; then, the MSD is not formed and the available energy of the jet is high [44]. When the stand-off distance increases, the MSD and oblique shock waves form into the kerf too; then, the loss in kinetic energy and momentum is increased and the dross removal is negatively affected. Furthermore, if the kerf width is reduced, a smaller amount of gas penetrates into the kerf and the MSD is stronger, reducing the available kinetic energy of the gas. In order to achieve good cut kerfs, sometimes the focus position is located above or underneath the surface of the workpiece to enlarge the kerf and increase the mass flow entering into the kerf.

Man et al. pointed out that the smaller the nozzle diameter, the better the flow field into the kerf and the higher the amount of molten material removal [44].

The results obtained by Ketting and Olsen [53] confirmed the assertions of Zefferer et al. [45]. They noticed an improvement in cutting results when the assist gas is moved away with regard to the laser beam. When the overlap of the assist gas jet with regard to the cutting front is at its maximum, the position of the boundary layer separation lies deeper, therefore, cutting quality and efficiency are maximized [43]. This is due to the higher entry of mass flow into the kerf (see Section 4.3).

**Figure 12:**
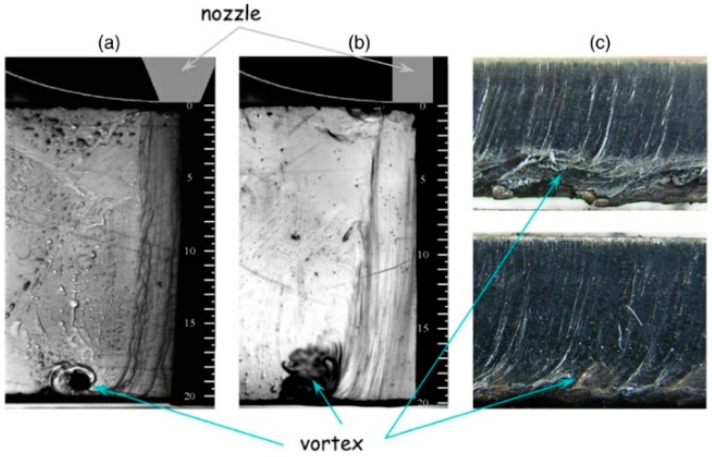
Vortex formed in the exit of the cut kerf using (**a**) converging, and (**b**) cylindrical nozzles. (**c**) Influence of their presence in the cutting quality during laser cutting of 20 mm thick mild steel using an oxygen jet (Supplying pressure *p* = 0.6 bar). Reprinted with permission from [37]. Copyright 2008 IOP Publishing.

Horisawa et al. [49,51] showed that after the boundary layer separation, recirculation zones (or vortices) appear along the cutting front (see Figure 12). These vortexes can trap some oxygen from the atmosphere and produce the undesirable oxidation of the cutting front during laser cutting using inert gases. On the other hand, they can trap nitrogen from the atmosphere and reduce the cutting performance during reactive laser fusion cutting because reduction in oxygen purity strongly affects the cutting process. Vortex formation in the lower part of the cutting edge was also detected by means of CFD-simulations and flow visualization into a simulated kerf [37]. Vortices were seen to be more pronounced when a cylindrical nozzle is used instead of a converging one. They pointed out the influence of these vortices on the dross attachment during low pressure reactive fusion cutting and proposed the utilization of an annular nozzle with an auxiliary gas (working at pressure higher than a minimum) to avoid vortex formation.

LaRocca pointed out that boundary layer separation and presence of vortexes in the bottom of the kerf makes assist gas more susceptible to ionization [32]. This is due to higher residence times, and contamination of the assist gas into the kerf (contamination due to vaporized species, and ejected particles from the cutting front).

### 3.4. Interactions of the Assist Gas with the Molten Material

There are not many works devoted to the visualization of laser cutting and the interactions between the assist gas and the molten material. The inherent difficulty of filming or modelling a hot melt moving at high speed into a small slot make it an arduous task.

Arata et al. [54,55] using X-Rays carried out the first studies on the interaction of the assist gas with the molten material into the kerf during reactive laser cutting of mild steel. They found that the striations could be caused by cyclical combustions. They also found that the movement of the molten material along the cutting front is almost cyclical. Hot streams of molten material from the upper part of the cutting front move downwards, but also spread laterally while they are getting colder. Presumably, the combination of the assist gas and the recoil pressure predicted by Semak and Matsunawa, due to the evaporation of molten material on the cutting front, causes this lateral spread [56]. If this molten material solidifies before being completely removed from the cutting edge, cuts with poor quality and a large HAZ are obtained. This can occur during laser cutting of materials, such as ceramics or aluminum and alloys, due to the large viscosity of the molten material. In the case of laser cutting of mild steel with oxygen, the reduced viscosity and surface tension induced by the formation of oxides avoids the presence of this resolidified layer to a large extent.

**Figure 13:**
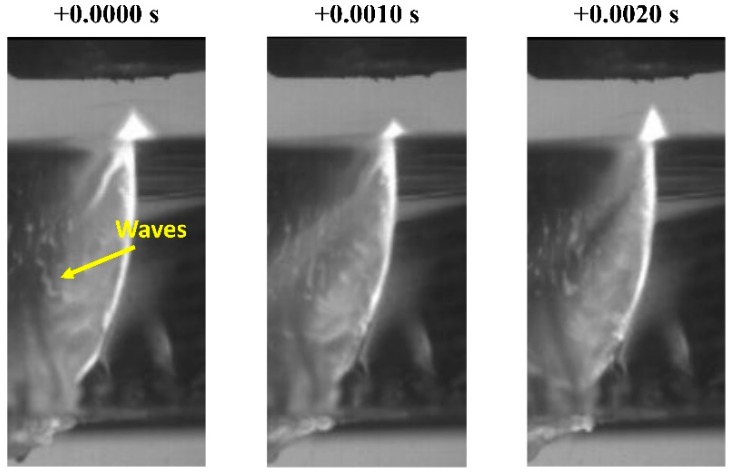
Waves on the molten material flowing along the cutting edge during laser cutting of glass (Processing conditions: Laser power *P* = 1400 W, cutting speed *v*_c_ = 1400 mm/min, conical nozzle, nozzle diameter *d* = 2 mm, stand-off *Z* = 1.5 mm, assist gas: argon, supplying pressure *p* = 8 bar). Reprinted with permission [57]; Copyright 2011 IOP Publishing.

Riveiro et al. [57] showed that some instabilities or waves are formed on the molten material while moving along the cutting front and cutting edge during laser cutting of glass (see Figure 13). The intensity of these waves is observed to be dependent on the assist gas pressure; however, this relationship is not linear because it depends on the laser power which ultimately determines the temperature and viscosity of the melt. The same waves were observed during laser cutting of Rose’s alloy [58].

The origin of these waves can be traced to interfacial instabilities between the gas and the melt flows. These waves are probably a consequence of Kelvin–Helmholtz instabilities produced by the interaction of the high-speed assist gas stream adjacent to the molten material [59]. According to Asali and Hanratty [60], these waves originate from the growth of small disturbances on the liquid film. Their growth is a result from an imbalance between the stabilizing effect of surface tension and the destabilizing effects of inertia, the component of the surface shear stress in phase with the wave slope, and the component of the surface pressure in phase with the wave height.

The formation of these waves could be the reason for the formation of some spray inside the kerf. Gross and O’Neill [61] used computer simulations to demonstrate that waves are formed in the melt, and that some parameter ranges exist where the melt film interacts violently with the applied gas jet. Furthermore, Kaplan [62] showed that the presence of these waves on the cutting front produces peaks on the local absorptivity which can lead to hot spots on the cutting front. These hot spots can locally produce large recoil pressures; however, more research (that is beyond the scope of this paper) on this topic is required to properly understand the role of these waves in laser cutting.

**Figure 14:**
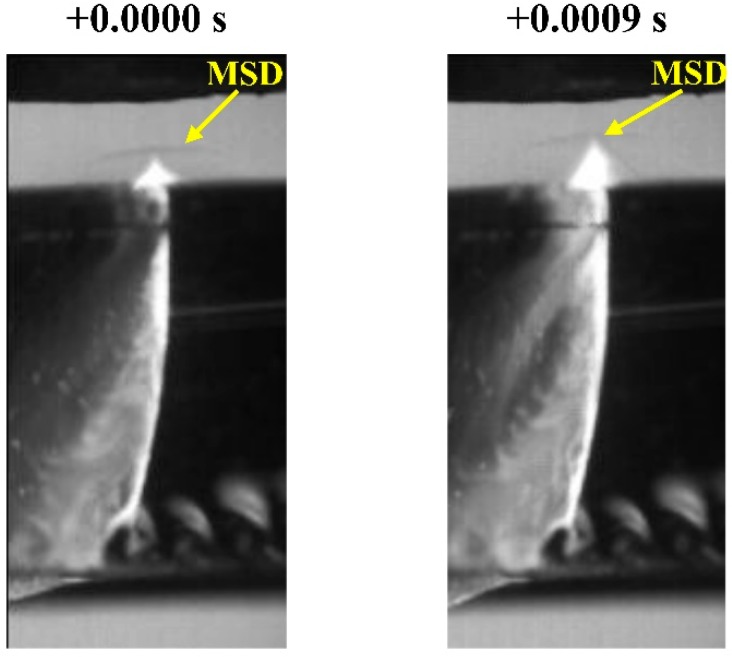
Displacement of the normal shock MSD due to the plume of ionized material emerging from the cut kerf (Processing conditions: Laser power *P* = 1400 W, cutting speed *v*_c_ = 1000 mm/min, conical nozzle, nozzle diameter *d* = 2 mm, stand-off *Z* = 1.5 mm, assist gas: argon, supplying pressure *p* = 8 bar). Reprinted with permission [57]; Copyright 2011 IOP Publishing.

An interesting phenomenon is the non-stationary character of the MSD [57]. Oscillations of the plasma plume emerging from the cutting kerf produce variations in the position of the MSD, as depicted in Figure 14. During these experiments, the plume of ionized material and the MSD were observed to oscillate with a frequency around 680 Hz. The influence of this oscillation on the gas flow pattern into the kerf, and on the flow of molten material remains unclear and should be investigated.

Tani et al. [63] developed a theoretical model to predict the dross adhesion in laser cutting. Results suggested that a minimum ejection speed of the molten material should be reached to overcome surface tension and frictional losses at the bottom of the kerf. A critical speed of 2500 mm/s was calculated for cutting steel plates (2, 3, 4 mm in thickness) with oxygen (*p* = 2 bar) as assist gas. Riveiro et al. also studied the formation of dross on the cutting edge [57]. The melt film formed along the cutting edge, and depicted in Figure 13; Figure 14, is responsible for the formation of dross. If the Weber number (ratio of the fluid’s inertia to the counteracting capillary pressure) for the melt reaching the lower cutting edge is close to the unit:

(10) $$\mathrm{We}=\frac{\rho_{m}\left(T\right)U_{m}^{2}\left(T\right)t_{m}}{\sigma_{m}\left(T\right)}\approx 1$$

the tendency to dross formation is high (where ρ_m_, *U*_m_, *t*_m_, and σ_m_ are, respectively, the density, velocity, thickness, and surface tension of the melt). In general, the lower the Weber number, the higher the tendency of dross formation. As pointed out by Schulz et al. [64] in a review on the advances in fundamental physical modeling and process monitoring of laser cutting, and by Riveiro et al. [57] after visualizing the cutting process by high speed photography, the confinement of the molten material into the cut kerf would avoid its lateral spread, and dross would not be formed. The use of off-axis nozzles (confinement of the melt by the quasi-normal impact of the assist gas jet on the cutting front), or other flow arrangements could be a solution to reduce the spread of the melt flow.

Observation of high power (30 kW) fiber laser cutting of very thick plates of carbon and stainless steel (300 mm) was carried out by Tamuri and Yamagishi [65]. From the combination of attenuated images of the cutting process, they were able to identify humps of molten metal that were generated by the aerodynamic interaction of assist gas flow with molten steel.

## 4. Proposals to Avoid Unsuitable Aerodynamic Interactions

### 4.1. Supersonic Nozzles

Kamalu and Steen highlighted the necessity of novel nozzle designs to obtain gas jets free of shock waves [22]. An alternative to typical converging nozzles are the converging-diverging nozzles, so called de Laval nozzles (in honor of the Swedish engineer Gustaf de Laval who developed the nozzle). Semrau and Tonshoff [66] pointed out that perfectly expanded flow exhausted by de Laval nozzles ensures the total conversion of supplying pressure into kinetic energy, and jets free of shocks and with a homogenous pressure distribution are obtained. Velocity of jets emerging from them can exceed the sonic speed, i.e., *M* > 1. This large velocity provides jets with large kinetic energy, which can be used to remove a large quantity of molten material by the mechanisms outlined in Section 2. These nozzles have a converging-diverging internal geometry, and are designed to isoentropically expand the assist gas up to the ambient pressure. Perfectly expanded jets are only obtained for an exact design pressure, which is determined by the ratio throat/nozzle exit areas [67]. Any deviations from the design pressure will cause a deterioration of the flow. Underexpanded free jets exhausted by de Laval nozzles show an MSD at a location with regard to the nozzle exit determined by the following empirical formula [68]:

(11) $$\frac{x}{D}=\sqrt{\frac{\gamma }{2}\frac{p_{e}}{p_{b}}M_{d}^{2}}$$

being: *D* the nozzle exit diameter, *p*_e_ the exit pressure; *p*_b_: back pressure (in general, the back pressure is equal to the ambient pressure, i.e., *p*_b_ = *p*_a_), and *M*_d_ the design exit Mach number.

Free jets exhausted by a converging-diverging nozzle are almost uniform and parallel for longer distances (up to several millimeters) than that exhausted by a converging nozzle. This increases the possible stand-off distance, which is particularly important to prevent optics and nozzle damage.

Maximum thrust is higher when using de Laval nozzles. Semrau and Tonshoff [66] noticed two advantages of using coaxial de Laval nozzles during reactive fusion cutting: an increase of the oxygen mass transport to the cutting front (which releases a higher quantity of heat to the process), and an increase in the flow rate into the kerf (which leads to the complete removal of molten material from the cutting front).

**Figure 15:**
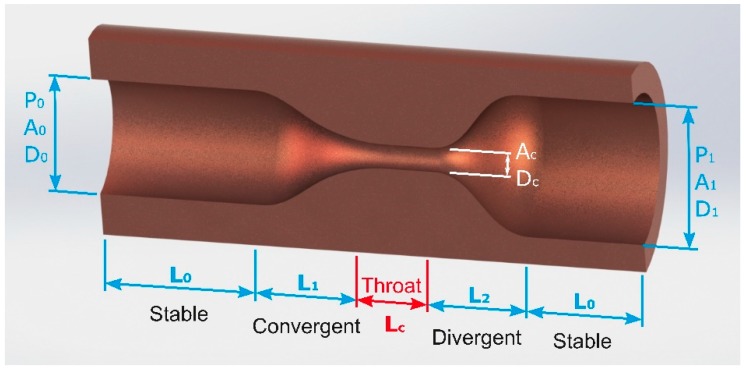
Scheme of a converging-diverging nozzle showing the different sections which compose the nozzle. Reprinted with permission from [69]; Copyright 1997 Elsevier.

Man et al. [23] described the steps involved into the design of supersonic nozzles, and studied the characteristics of such jets from numerical simulations and shadowgraph visualizations. They are mainly composed of four sections, namely, a stable, convergent, throat and diverging sections, as depicted in Figure 15.

The stable section is designed in order to transform the supplied assist gas into a uniform and non-turbulent flow. The diameter *D*_0_ (or area *A*_0_) of this section is dependent on the throat diameter *D*_c_ (or area *A*_c_). The convergent section is designed in order to accelerate the assist gas flow, keeping it uniform and parallel. This section is dependent on the convergent/throat area ratio imposed by the Equation (12) (see reference [24] for more details),

(12) $$\frac{A_{0}}{A_{c}}=\frac{M_{c}}{M_{0}}{\left(\frac{1+\frac{\gamma -1}{2}M_{0}^{2}}{1+\frac{\gamma -1}{2}M_{c}^{2}}\right)}^{\frac{\gamma +1}{2\left(\gamma -1\right)}}$$

where *A*_0_, *A*_C_ are the cross section area at the inlet and throat, respectively, and *M*_0_, *M*_C_ are the Mach number at the inlet and throat sections, respectively.

Crown [70] proposed that the converging curvature can be approximated by two arcs. The gas flow is transformed from subsonic into supersonic through the throat section. Finally, the divergent section allows accelerating the flow up to the operation Mach number at the exit. The exit area is given by Equation (13):

(13) $$\frac{A_{1}}{A_{c}}=\frac{M_{c}}{M_{1}}{\left(\frac{1+\frac{\gamma -1}{2}M_{1}^{2}}{1+\frac{\gamma -1}{2}M_{c}^{2}}\right)}^{\frac{\gamma +1}{2\left(\gamma -1\right)}}$$

where *A*_1_ and *M*_1_ are the cross section and Mach number at the outlet.

For this kind of nozzles, the mass flow is limited by the area of the throat and can be calculated by Equation (14):

(14) $$m=\rho_{c}V_{c}A_{c}$$

where ρ_c_ and *V*_c_ are the density, and velocity of the assist gas at the throat. Calculation of the inner profile of this section can be accomplished by the method developed by Foelsch [71].

Duan et al. [72] largely studied the flow pattern into the kerf using supersonic jets. Gas flow into the kerf was seen to be independent of the stand-off due to the non-radial expansion of the jet. Man et al. [44] observed that a higher amount of gas penetrates into the kerf as compared to the processing assisted by converging nozzles. Leidinger et al. [73] showed that pressure distribution and Mach number on the workpiece were insensitive to stand-off variations when a de Laval nozzle is used.

Man et al. [44] also found the generation of a detached shock wave ahead of the cutting front as a consequence of the utilization of a coaxial de Laval nozzle. The position of this shock wave was evaluated and Mach number and pressure distribution were obtained from the nozzle to the kerf exit. Pressure was found to be increased and Mach number decreased after crossing the detached shock wave. Gas jet is expanded after this detached shock. An oblique shock wave impacting on the cutting front was detected which increases the pressure in the flow. This could cause boundary layer separation, which reduces shear stress in the cutting front and leads to poor cutting quality. Under this kind of assist gas supplying, gas velocity and pressure exhibit a non-linear distribution that could result in non-linear shear stress distribution along the kerf.

If the kerf width is decreased, the flow pattern into the kerf deteriorates because some oblique shock waves are formed in the kerf inlet. An MSD can be formed if the kerf width is extremely reduced [44]. Under this conditions, viscosity effects become stronger.

Cutting trials using supersonic coaxial nozzles were made on several different materials. Mukherjee et al. [74] tested the performance of an own-design supersonic coaxial nozzle during laser cutting of 1 inch thick kiln-dried basswood sheets. Up to a 50% of improvement in cutting speed, as compared to the cutting with a standard nozzle, and for a constant laser power of 1560 W was obtained. However, cut quality was not substantially improved. Semrau and Tonshoff [66] empirically compared the performance of a de Laval nozzle with regard to other nozzle designs, and in particular with a converging arrangement. Higher performance of the converging-diverging nozzle in terms of cut quality and speed was pointed out during cutting 2 mm steel sheets. Other works, such as that performed by Boutinguiza et al. [75], also emphasized the possibility of attaining a higher cutting speed when a converging-diverging nozzle is used instead of a conical coaxial arrangement working at the same gas pressure during CO_2_ laser cutting of slate tiles.

Supersonic nozzles for laser cutting have two main drawbacks. First, any deviation of the supplying pressure from the theoretical design pressure produce jets similar to those exhausted by converging nozzles. In this sense, deviations >40% from the designed pressure severely deteriorate the flow pattern into the kerf, enhancing the sensitivity to stand-off variations [44]. Second, fabrication of de Laval nozzles is an arduous task. Nozzles in the micrometer range are required to produce small jets and then maintain the choking (see Section 3.2) under acceptable values. Such reduced dimensions make the machining of the inner converging-diverging profile complicated. Micro-milling or electrical discharge machining (EDM) are the most feasible manufacturing techniques of these nozzles. It should be noted that the final internal surface roughness in these nozzles have a strong impact on its performance. The inner surface roughness in the nozzle can led to the formation of multiple weak shocks along its divergent part, which can bring the nozzle performance loss up to 18% [76].

### 4.2. High Pressure Gas Laser Cutting with Nonreactive Gases

**Figure 16:**
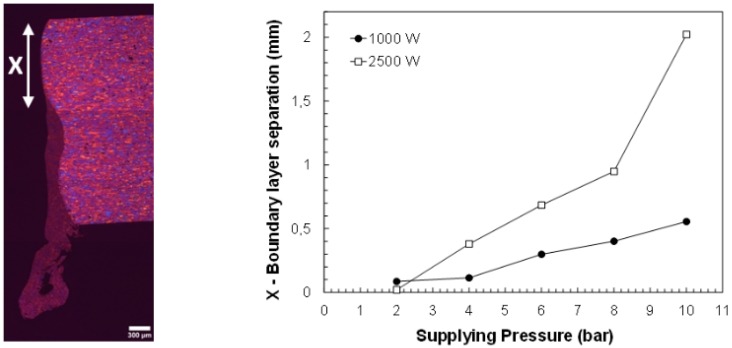
Detachment of the boundary layer as a function of the assist gas pressure during laser cutting of aluminum-copper alloys. Reprinted with permission from [30]; Copyright 2010 Elsevier.

Different authors noticed a lower position of the boundary layer separation, along the cutting edge, when the assist gas pressure is increased. Rand [77] using CFD analyses corroborated this result previously found by Zefferer et al. [45] using Schlieren methods, and by Horisawa et al. [52] using Schlieren methods and CFD analyses. Then, the upper low roughness region (observed in Figure 11a) is larger. Riveiro et al. [78] experimentally observed the enlargement of this region with the supplying gas pressure during CO_2_ laser cutting of 2024-T3 alloy (see Figure 16). As observed, the increment is dependent on the laser power, because this parameter controls the temperature of the molten material and, consequently its viscosity as well.

Rand et al. [79] numerically showed that underexpanded jets at high pressures propels the molten material towards the cutting edges. Therefore, dross can be easily formed in the bottom of the cutting edge under some processing conditions, resulting in poorer cut quality.

Cut quality during inert laser cutting can be substantially improved by increasing the supplying pressure of the assist gas [19]. Cutting speed is also significantly improved. However, an increment of the gas pressure during reactive laser fusion cutting (using air or oxygen) produces poor cut quality due to excessive burning effects. It was noticed that pure oxygen gas pressure would range from *p* = 0.75 to 2 bar during CO_2_ laser cutting of 3 mm mild steel (laser power *P* = 1500 W) to obtain good quality cuts.

Anon [80] showed the possibility to cut sheet metal of up to 3 mm thick using inert gases at high pressure. Also, Bierman et al. [81] processed aluminum alloys at high-pressure and examined cuts from a quality point of view. Same approach was conducted by the group of Prof. Olsen [53,82] during cutting pure aluminum and an Al-Mg alloy. Dross free cuts or with small and easy removable dross were obtained during cutting sheets with 1 to 2 mm in thickness, using N_2_ at pressures from 5 to 15 bar. Results were improved if the assist gas jet is moved away with regard to the laser beam, as will be reviewed in Section 4.3. Using this approach, cuts with an acceptable quality for most of the applications can be obtained, but the gas consumption is considerably increased.

### 4.3. Off-Axis Nozzles

Most of work performed on the role of gas dynamics in laser cutting was performed using only nozzles coaxial with the laser beam. Some authors pointed out that off-axis nozzles increase cutting efficiency and quality. The superior performance relies on different causes. Arata et al. [55] proposed the use of an off-axis nozzle in tandem with a coaxial nozzle. After cutting trials on stainless steel and titanium samples they verified the improvement in cutting quality and the reduction in dross height (up to 1/3 as compared to the utilization of a single coaxial nozzle). Also, Tomie et al. [83] used an off axis nozzle in combination with a coaxial one during CO_2_ laser cutting of 6 mm alumina. The jet exhausted by the off-axis nozzle, which was positioned behind the coaxial one, was directed to the bottom of the edge of the kerf in order to remove the clinging dross. Dross free cuts were obtained by means of this arrangement. Chryssolouris and Choi [84] proposed the utilization of the off-axis nozzle to remove the molten material from the kerf, and the coaxial nozzle was only used to protect the lens. They addressed additional parameters using off-axis nozzles, namely, nozzle angle, supplying pressure, nozzle/workpiece distance, and jet targeting point (or distance between centers of coaxial and off-axis nozzles). They experimentally tested the influence of these parameters on the cutting depth and compared the results with those obtained using only a coaxial jet. Using the off-axis nozzle the cutting depth was increased up to a 20% as compared to the coaxial one, because of the increase in the momentum transfer to the molten layer. They also noticed that too large nozzle/workpiece distances decrease the cutting depth because the jet spreads and less mass flow penetrates into the kerf. On the other hand, the jet targeting point is also a critical parameter because if the jet is not entirely sweeping the cutting front, the cutting depth is decreased. Finally, they noted that using low off-axis nozzle/laser beam angles improves the removal of molten material because the momentum transfer is increased.

Ilavarsan and Molian [85] followed the previous experimental approach. Using an off-axial cylindrical nozzle, in conjunction with a converging coaxial one, the cutting speed during reactive gas laser fusion cutting of carbon steel, and 304-stainless steel is substantially increased for thicker plates (plates with thicknesses in a range of 2–4.5 mm); however, the off-axial assist gas did not have any significant effect when cutting thin plates (<2 mm). Hsu and Molian [86] used an analogous experimental setup during CO_2_ laser cutting of 6.35 mm thick AISI304 stainless steel plates. They found a decrease of the roughness in the cutting edge, and a reduction in the resolidified layer; moreover, it is feasible to obtain dross free cuts. The material removal rate, and the amount of dross was found to decrease with the off-axis nozzle/laser beam angle. Nevertheless, Schuöcker [87] found that at low angles cut quality decreases in the bottom of the cut. This was attributed to the lower temperature of the melt and the turbulent regimen of the gas in this region.

**Figure 17:**
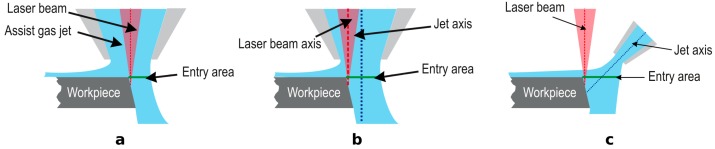
Schematic view of the (**a**) on-axis cutting process, (**b**) off-axis cutting process with the assist gas jet impinging normally to the workpiece, and (**c**) when the assist gas jet forms an angle with the laser beam.

Using an off-axis nozzle with the jet normally impinging on the workpiece, as depicted in Figure 17b, Ketting and Olsen [53] found that the range of parameters which lead to dross free cuts was expanded during cutting trials of stainless steel and aluminum samples. The explanation relies on the higher amount of gas entering into the kerf. Chen et al. [46] pointed out that a higher mass flow entering into the kerf increases the molten material removal. CFD simulations demonstrated that moving the nozzle backwards with regard to the laser beam promotes an increase of the viscous and pressure forces on the cutting front [79]. Thus, the first advantage related with the processing assisted by an off-axis nozzle is the increment of the mass flow entering into the kerf (see Figure 17b,c). Furthermore, these authors noticed that the sensitivity to variations of the focus position (a crucial parameter to obtain good quality cuts) decreases using an offaxis nozzle. Graaskov et al. [88] demonstrated the industrial implementation of this technique by using the on-line adjustment of a bending mirror.

Despite this experimental work, few efforts were carried out in order to understand the influence of using off-axis nozzles on the aerodynamics of the assist gas during the cutting process. Work performed by Brandt and Settles [89] entailed a serious approach to understanding the key issues involved in the utilization of an off-axis nozzle. Using the Schlieren technique (see reference [90] for more information about this technique), they visualized the flow field into a simulated kerf.

**Figure 18:**
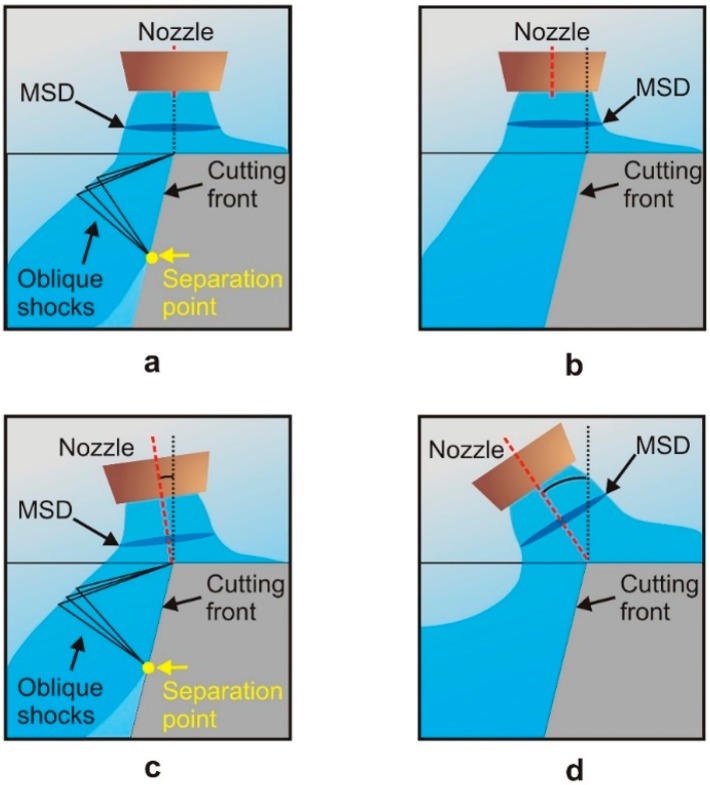
A schematic view of the aerodynamic interactions for (**a**,**b**) different nozzle positions, and (**c**,**d**) different nozzle tilt angles. The arrow in (**a**) and (**c**) denotes the boundary layer separation. Adapted with permission from [89]; Copyright 1997 AIP Publishing.

They studied the effect of the displacement of the gas jet with regard to the laser beam (see Figure 18a,b). They noticed that as the jet is moved towards the leading edge of the cutting front (as occurs in conventional laser cutting and depicted in Figure 18a), the pattern of compression shock waves formed after being reflected in the jet boundaries coalesces into an oblique shock wave. This wave has enough strength to produce the boundary layer separation (formed by the assist gas with the cutting front) when impacts on the cutting front, as depicted in Figure 18a. Therefore, when nozzle is moved away from the laser beam as shown in Figure 18b, the strength of the interaction shock wave with the boundary layer decreases. This creates a uniform flow throughout the whole cutting edge as seen in Figure 18b.

**Figure 19:**
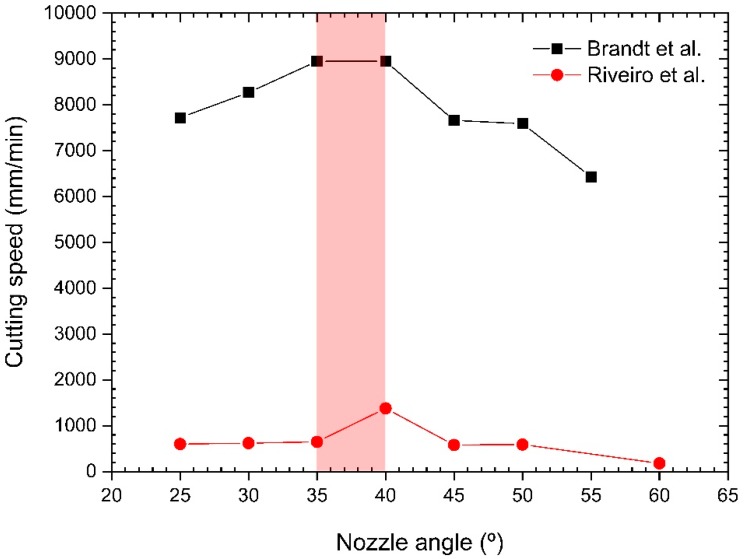
Maximum cutting speed as a function of the nozzle angle for a sonic exit nozzle [89] and for a supersonic exit nozzle [91] during CO_2_ laser cutting of 3 mm mild steel and 3 mm Al-2024-T3 alloy respectively. Optimum nozzle angles range from 35° to 40° with regard to the laser beam.

On the other hand, they investigated the influence of the nozzle angle on the flow characteristics of the assist gas into the kerf. They noted that boundary layer separation appears for small nozzle/laser beam angles and the flow pattern into the kerf is similar to that generated for coaxial nozzles (as depicted in Figure 18c). However, nozzle/laser beam angles larger than 20° produce a uniform flow field inside the kerf, as seen in Figure 18d, and the boundary layer separation is not produced. Under these conditions, the flow must be turned by an angle to flow along the cutting front. This turning cancels the expansion waves that produce the compression waves, which induce the oblique shock impinging on the cutting front. As a consequence, the strength of the oblique shock wave decreases, and is not able to produce the boundary layer separation. Optimum angle range that sensibly increases the cutting speed was found to be 35°–40° by means of cutting trials. A similar range of angles was found during fiber laser cutting of 2024-T3 alloy [91] (see Figure 19).

The work by Quintero et al. [50,92] expanded the results obtained by Brandt and Settles [89] to the utilization of an off-axis de Laval nozzle. They presented a comprehensive revision of the main factors required to optimize the efficiency of a cutting head assisted by an off-axis supersonic nozzle. Supersonic nozzles with 15 mm of supersonic length were designed to assure a uniform jet for cutting thick sectioned materials. They also noticed that the impinging point of the jet was a crucial parameter [93]. Visualization of the jet by means of Schlieren technique into a simulated kerf and the comparison with cutting experiments demonstrated the transformation of the jet into the kerf from turbulent to laminar regimen when the impingement point varies. When the jet impinges on the leading edge of the cutting front, a detached shock wave is formed just in the entry of the kerf. This shock promotes the detachment of the boundary layer into the kerf. Moving the jet away with regard to the laser beam promotes the reduction of the strength of the shock wave, but an oblique shock wave impinging on the cutting front is formed, also producing the detachment of the boundary layer. However, there exists a position in which the oblique shock wave does not impinge on the cutting front and a boundary layer is developed throughout the whole cutting front. Under these conditions, the assist gas flow correctly sweeps the molten material. Then, high quality cuts can be obtained in cutting ceramics [93] or aluminum alloys [78,94,95]. The improvement in cutting speed and quality during laser cutting of 2024-T3 sheets was dependent on the nature of assist gas [95]. Cutting speed and quality are maximum using a supersonic jet of argon. Utilization of nitrogen, compressed air, or oxygen produce oxides and nitrides which affect cut quality and cutting speed due to an increment in the melt viscosity.

Table 2 summarizes the optimal conditions for the off-axis nozzle required for obtaining good quality cuts as proposed by different authors in the most relevant performed works.

### 4.4. Annular (Ring) Nozzles

Annular (or ring) nozzles were proposed to obtain assist gas jets with a more favorable shock wave structure for laser cutting. Masuda et al. [97] performed an experimental study on the impingement of this kind of jets on a workpiece by means of pressure measurements and visualization of the jet with Schlieren and Shadowgraph techniques. The observed flow pattern is more complex than in converging or de Laval nozzles. They pointed out the large influence of the stand-off distance. Stand-offs higher than a critical value make the streamlines issuing from the inner edge of the annular nozzle converge at a point. Then, high pressures in the center of the jet act on the surface of the workpiece. The high pressure region is confined to a narrow region on the center of the jet. This implies that the region of high pressure can penetrate into smaller kerfs. The pressure distribution on the workpiece for different stand-offs is shown to be more uniform as the pressure of the annular jet is increased. However, a fine adjustment of the stand-off is required because the pressure acting on the workpiece rapidly decays from the optimal stand-off distance.

**Figure 20:**
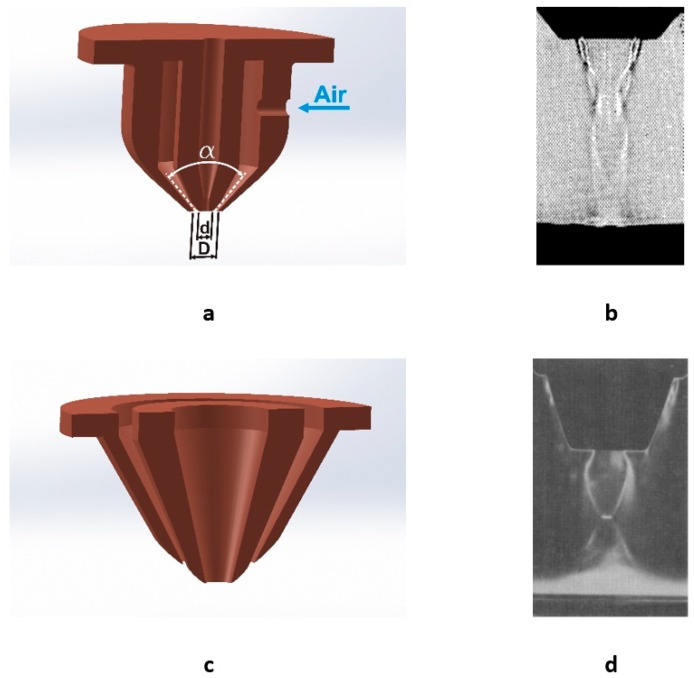
(**a**) Parameters needed to define the nozzle contour in annular nozzles used for laser cutting and (**b**) shadowgraph of the free jet exhausted by an annular nozzle defined by *d*/*D* = 0.8, and α = 40° at an assistant pressure of *p*/*p*_a_ = 9. (**c**) Coanda nozzle, and (**d**) Schlieren image of an impinging jet ejected by this kind of nozzle. Reprinted with permission from [97,102]; Copyright 1990 The Japan Society of Mechanical Engineers, 1992 SPIE.

Masuda and Nakamura [98] noticed that nozzle contour is a critical parameter on the nozzle performance. Parameters defining the nozzle contour and influencing the flow were found to be the inner to outer diameter ratio *d/D*, and the angle of ejection *α* (see Figure 20a,b). Large *d/D* ratios, and α angles (*d*/*D* = 0.8 and α = 40°) produce very high pressures on the surface of the workpiece.

Annular nozzles can control the flow structure of a jet exhausted by sonic nozzles. Masuda and Moriyama [99] observed the reduction in the MSD diameter created in the inner jet exhausted by the converging nozzle when the pressure of the annular jet is increased. In general, the shock wave structure of the coaxial jet is improved if the annular nozzle is ejected with some angle with regard to the laser beam.

Annular nozzles were also proposed to control the purity of the assist gas. As noted by Powell et al. [100], a 1% reduction in the purity of oxygen can produce a 25% reduction in cutting speed due to the reduction in energy supplied to the cutting front. Annular nozzles were used by Thomassen and Olsen [101] during laser cutting of steel. They pointed out the possibility of the annular jet to retain the oxygen at the interaction zone. O’Neill and Steen [40] proposed the utilization of an annular nozzle to control the purity of an oxygen jet into the cut kerf. The results suggest an increment of the oxygen concentration into a simulated kerf. Cutting trials on mild steel confirmed the benefits in order to cut thicker sections.

Coanda nozzles can have some degree of success to avoid the formation of the MSD [102]. The Coanda nozzle, depicted in Figure 20c,d, is similar to the annular one. The difference is found in the rounded contour of the external wall of the central nozzle in the Coanda nozzle. Due to the Coanda effect (see reference [103] for more information on this effect), the gas follows this contour and the central jet experiences a local pressure higher than the atmospheric. This produces the breakdown of the MSD at pressures at which the central nozzle, on its own, would form an MSD due to the low value of the *p*_e_/*p*_0_ ratio. These nozzles can exert high gas pressures at stand-offs of 4–5 mm [102].

### 4.5. Off-Axis Supersonic Rectangular Nozzles

The most recent development in this field includes the utilization of supersonic rectangular nozzles non-coaxial with the laser beam. This approach improved the efficiency of the current assist gas injection systems, especially in terms of surface quality, cutting speed and gas consumption [104]. The roughness of the cut edge is reduced in a factor of 6.5, and the cutting speed increases by a factor of 2 as compared with conventional converging coaxial nozzles [104]; moreover, dross-free cuts with a negligible HAZ are obtained. In order to obtain such qualities these systems must be optimized. The relevant optimization parameters are: (1) nozzle inclination, and (2) impact point. As demonstrated by same authors, this optimization leads to a high confinement of the molten material close to the cutting front [104,105]. This is consequence of the lower choking experienced by the assist gas because its geometry matches that of the kerf.

## 5. Conclusions and Future Prospects

In this review, an attempt was made to survey main findings related to the role of the assist gas during laser cutting. The basic gas dynamics interactions are practically understood. The presence of normal and oblique shock waves, and their interaction with a boundary layer formed on the cutting front largely determine the cutting speed and quality. However, this review revealed some deeper questions that require further research, namely:

- Most of the research carried out in the past neglects the presence of a hot viscous film of molten material on the cut kerf. The understanding of the interaction between the assist gas and the molten material is vital because its interaction is the main issue in the removal of the melt. The unique technical challenges present in the process require CFD simulations or novel experimental methods.
- Novel nozzle designs or even new arrangements to extract the molten material from the cut kerf in a most efficient way are required. This task should be economically viable because the conventional nozzles are easily manufactured and cheap in comparison with the rest of elements used in a laser cutting unit. These designs should be based on the previous point to correctly address the task of the nozzles.
- Nozzles are currently manufactured in mass. In the literature, no serious studies on the most appropriated materials and machining techniques for these parts are found. Furthermore, the influence of the finishing on the nozzle performance during laser fusion cutting has not been addressed.

Nowadays, laser sources emitting high-power and high quality beams are ready to be currently used by the industry; however, the potential of new high-brightness laser sources (e.g., fiber or disk lasers) available for laser cutting is not completely exploited due to the inefficient removal of molten material by the gas jets generated by conventional nozzles. Combination of current high-power laser sources and more efficient nozzles will lead the process of cutting with photons as the standard machining technique for the 21st century.

## Figures and Tables

**Table 1:** Processing parameters related to the dynamics of the laser cutting processes [9].

Laser Beam	Beam Guidance	Assist Gas	Transport Properties	Material
Wavelength	Focusing element	Chemical composition	Cutting speed	Absorptance
Beam diameter	Focal length	Density		Density
Beam mode	Focus position	Viscosity		Viscosity
Beam parameter product		Assist pressure		Melting point
Spatial intensity distribution (Beam profile)		Nozzle geometry		Evaporation point
Polarization		Nozzle diameter		Specific heat capacities
CW/Pulsed mode		Nozzle alignment		Thermal diffusivity
Pulse frequency		Stand-off distance		Latent heat of fusion
Duty cycle				Latent heat of evaporation

**Table 2:** Optimal conditions for the off-axis nozzle required for obtaining good quality cuts as proposed by different authors.

Nozzle Type	Nozzle Diameter (mm)	Angle Off-Axis Nozzle/Laser Beam (°)	Supply Pressure (bar)	Reference
–	–	50	2.5	[55]
Converging (Oval shape)	5.5	35	4.8	[84]
Cylindrical	1.1	40	2.6–4.6	[85]
Cylindrical	1.1	38–40	>3.1	[86]
Cylindrical	1.1	40	3.3–3.9	[96]
Converging	1.5	35–40	6.2	[89]
Laval	1.7	45–55	8	[92]
Laval	1.7	45–55	8	[94]

## Nomenclature

*a*	Adimensional function
*A*_0_	Cross section area at the inlet
*A*_1_	Cross section area at the outlet
*A*_c_	Cross section area at the throat
*b*	Adimensional function
*C*_f_	Local skin friction coefficient
*d*, *D*	Nozzle exit diameter
*f*	Friction coefficient; focal length
*F*_0_	Force exerted by the gas on the cutting front due to static pressure
*F*_n_	Normal component of the dynamic force exerted by the gas on the cutting front
*F*_t_	Tangential component of the dynamic force exerted by the gas on the cutting front
*M*	Mach number
*M*_0_	Mach number at the inlet
*M*_1_	Mach number at the outlet
*M*_c_	Mach number at the throat
*p*	Pressure of the assist gas
*p*_0_	Reference pressure; stagnation pressure
*p*_a_	Ambient pressure
*p*_b_	Background pressure
*p*_e_	Pressure just at the exit of the nozzle
*p*_g_	Reduced pressure
*P*	Laser power
*R*_a_	Average roughness
Re_g_	Reynolds number of the assist gas
*t*_m_	Melt film thickness
*T*	Temperature
*U*_g_	Velocity of the assist gas
*U*_m_	Velocity of the molten material
*v*_c_	Cutting speed
*V*_c_	Velocity of the assist gas at the throat of the de Laval nozzle
*w*	Kerf width
*x*	Distance along the centerline of the jet; distance along the cutting front or cutting edge
*x*_M_	Position of the Mach disk with regard to the nozzle exit
*Z*	Stand-off
γ	Heat capacity ratio (γ = *c*_p_/*c*_v_)
ρ_c_	Density of the assist gas at the throat of the de Laval nozzle
ρ_g_	Density of the assist gas
ρ_m_	Density of the molten material
σ_m_	Surface tension of the molten material
τ	Shear stress acting on the cutting front due to the assist gas
φ	Inclination of the cutting front
µ_g_	Dynamic viscosity of the assist gas
